# The promises and challenges of robotic-assisted surgery in regional Australia

**DOI:** 10.1007/s11701-026-03234-7

**Published:** 2026-02-18

**Authors:** Yung-Hsin Hsueh, Gavin J. Carmichael, Jessica Hanna, Kirk Underwood, Daniel Ng Ying Kin, Dongrong Situ, David Low, Mathew Jacob

**Affiliations:** 1 Department of Surgery, Grampians Health, Ballarat, Australia; 2 University of Melbourne, Melbourne, Australia; 3 Grampians Research Initiative (GRIT), Ballarat, Australia

**Keywords:** Robotic-assisted surgery, Rural surgery, Surgical training, Surgical sustainability, Australia, Health care delivery

## Abstract

This perspective examines the introduction of robotic-assisted surgery (RAS) in rural and regional Australia, using the experience of a single regional public hospital as a case exemplar. It explores potential benefits, implementation challenges, and broader implications for surgical equity, workforce sustainability, and health-system planning. Rural Australians face persistent disparities in healthcare access, surgical outcomes, and specialist availability. Robotic-Assisted Surgery offers superior visualisation, precision, and patient recovery compared to traditional approaches, but remains largely concentrated in metropolitan centres. The inequitable distribution of RAS risks exacerbating urban–rural health divides unless deliberately addressed. The paper synthesises emerging evidence on RAS implementation across specialties, including colorectal, thoracic, urology, and gynaecology. It explores workforce implications, economic modelling, and training infrastructure challenges in the context of regional deployment. The Ballarat program is profiled in detail, including procedure volumes, workforce upskilling, and local system impact. Key enablers and barriers to sustainable RAS integration are mapped, with reference to training models, policy frameworks, and funding structures. The introduction of RAS in regional centres supports decentralisation of complex surgical care, and attracting a modern surgical workforce in turn contribute to reducing geographic healthcare inequity. While promising in terms of clinical outcomes and economic value, challenges remain around cost, training accessibility, and long-term sustainability. National strategies, investment in regional training pipelines, and equitable funding models are critical to ensure RAS becomes a scalable and inclusive solution for rural surgical care in Australia.

## Introduction

Rural and regional Australia has long faced significant disparities in healthcare compared to metropolitan centres. People living outside major cities experience higher rates of hospitalisation and avoidable mortality, with rural Australians having 2–3 times the rate of potentially preventable hospital admissions compared to urban patients [1]. Rural patients have poorer access to specialist care; with only approximately 12% of surgical specialists practicing in these areas, despite approximately 29% of the population living in rural or remote Australia [2]. The lack of specialist surgical services compels many patients to travel extensive distances for complex minimally invasive surgery (MIS). Additionally, limited access to advanced surgical interventions, such as robotic-assisted surgery (RAS), exacerbates health inequalities, contributing to poorer outcomes for rural populations.

**Fig. 1:**
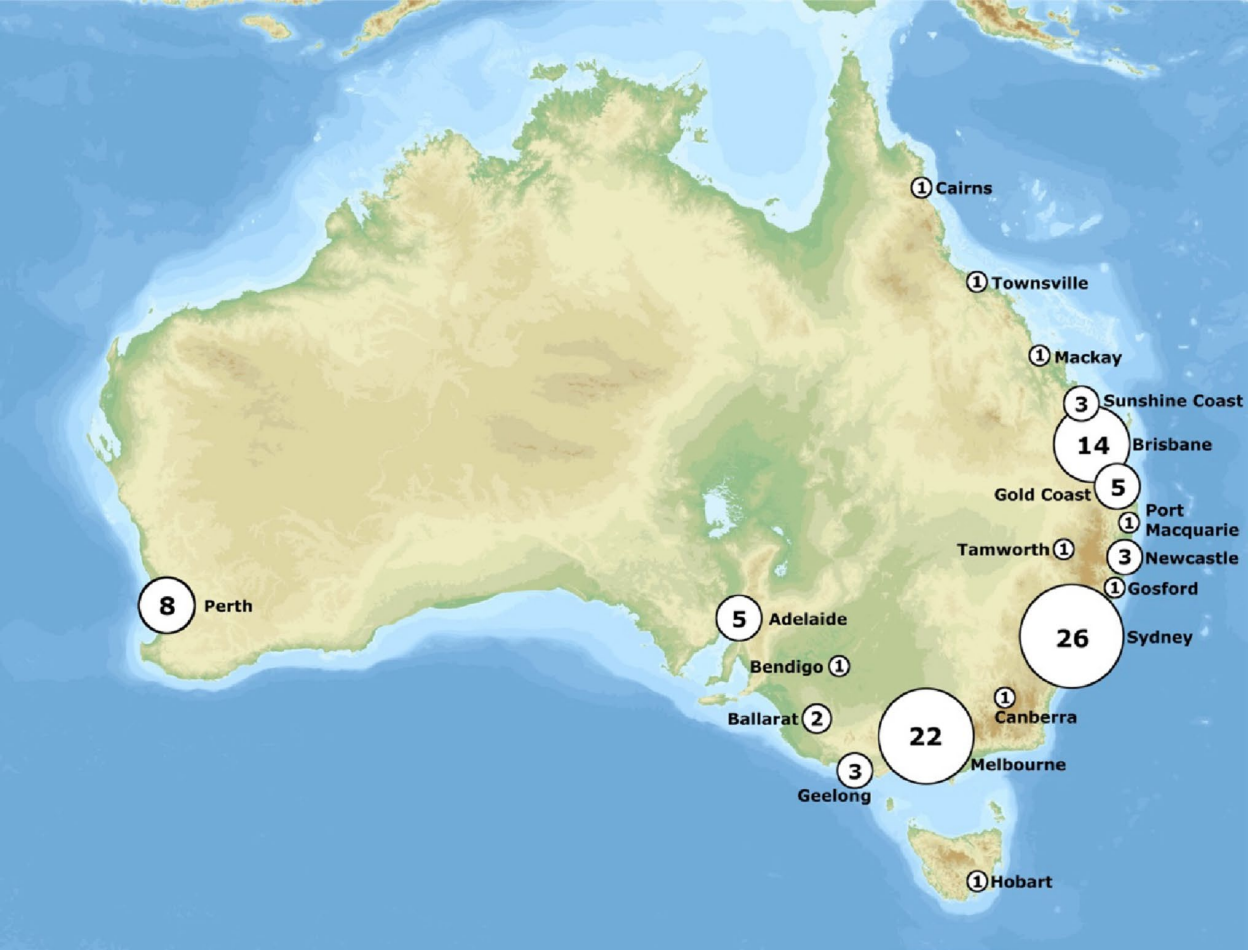
Intuitive Da Vinci^®^ Robotic Consoles in Australia. Adapted from: Thennicke. *Australia high resolution topography and bathymetry* [Internet]. Wikimedia Commons; [cited 2025 Jul 11]. Available from: https://commons.wikimedia.org/wiki/File:Australia_high_resolution_topography_and_bathymetry.png. Licensed under CC BY-SA 3.0, and from Intuitive Robot Data from January 1, 2022 to December 31, 2024. Melbourne: Device Technologies; 2025.

RAS is the largest paradigm shift in MIS since laparoscopic surgery, and offers numerous advantages including enhanced surgical precision, improved surgeon ergonomics, and superior visualisation, ultimately leading to better patient outcomes [3]. Since the development of surgical robotic platforms like the Da Vinci ^®^ System in 1999, North America has led their exponential integration into hospitals. Following suit, Australia and New Zealand have seen a significant increase in RAS adoption since its introduction in 2003. As of 2025 May, a total of 112 Da Vinci ^®^ platforms are now available among Australia (Fig. 1) and New Zealand. These platforms are mainly located in the private sector and based in metropolitan centres, with only 2 out of 26 public platforms across Australia located rurally or regionally [4, 5]. This highlights a problem that may see increased disparity in surgical care provided in rural and regional centres.

While RAS adoption remains limited within Australia’s public health system, the introduction of the first regional public robotic platform at Grampians Health – Ballarat Base Hospital in late 2022 provides a unique opportunity to examine implementation outside metropolitan centres. This paper presents a single-centre regional experience that examines the implementation of RAS in rural and regional Australia, using the experience of Ballarat as a detailed case exemplar to highlight real-world opportunities, constraints, and lessons for other regional centres.

## The promises of robotic-assisted surgery

### Enhanced access to advanced care

**Fig. 2:**
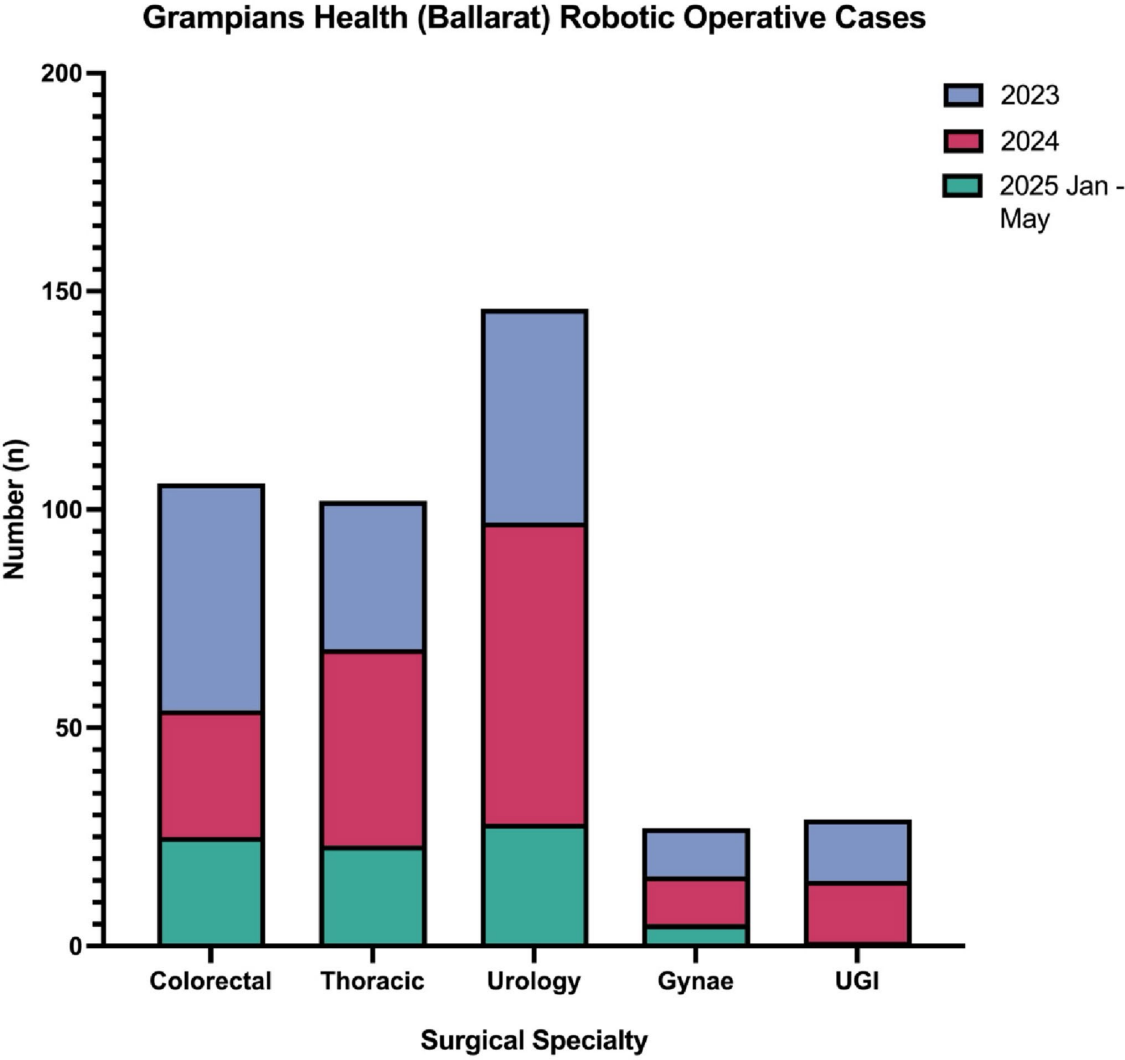
Ballarat Base Robotic Operative Cases breakdown in specialities (2022–2025)

Since 2023, Ballarat Base Hospital has joined a select group of public hospitals in Victoria offering robotic surgery, including The Peter MacCallum Cancer Centre, Barwon Health, The Royal Melbourne Hospital, and Austin Health. Importantly, Ballarat houses the only regional Victorian public robotic platform [4, 6]. Since installation of a Da Vinci^®^ system, Ballarat surgeons performed around 409 RAS procedures as of May 2025, including complex colorectal (26.16%), thoracic (24.40%), urology (35.70%), gynaecology (6.60%), and other general surgical cases (7.14%) (Fig. 2). The availability of RAS in regional centres facilitates decentralisation of health care and represents a step forward in reducing the urban-rural healthcare divide.

It is evident that patients are increasingly well-informed about health innovations and emerging medical developments. In regional areas, this awareness may motivate individuals to travel in search of advanced care when such services are not locally available. Facilitating timely access to complex procedures closer to home not only reduces the travel burden and enhances continuity of care for rural patients [7], but also benefits the broader healthcare system. However, early regional robotic programs may preferentially serve selected patient cohorts and referral pathways, meaning improvements in access may initially be uneven across populations.

### Improved patient outcomes

Robotic surgery enables a minimally invasive approach that overcomes the limitations of traditional laparoscopic and thoracoscopic surgeries by enhancing surgical visualisation, increasing precision, and providing greater dexterity. Furthermore, robotic systems allow for delicate tissue handling and precise suturing, which can be particularly beneficial in high complexity case-mixes such as deep pelvic or complex oncological procedures [8].These advantages have been associated with shorter hospital stays, reduced post-operative complications, and faster recovery times [9]; which may provide a long-term cost-benefit to the healthcare system.

In multiple surgical fields, meta-analyses show robotic approaches yield benefits over conventional techniques. In colorectal surgery, recent reviews found that for obese patients, robotic resection significantly shortened length of stay and reduced blood loss compared to laparoscopy [10]. In advanced rectal cancer, robotics also yielded a dramatically lower conversion rate to open surgery [11]. Benefits have been mirrored in urological surgery [12], with findings of shorter hospital stays, less blood loss, and fewer complications on average with robotics compared to open or laparoscopic approaches. Taken together, the quantitative literature supports that RAS in diverse specialties generally shortens hospitalisation, lowers intraoperative difficulty, and does not worsen complication rates. Much of this evidence originates from high-volume metropolitan centres with established robotic programs and may not be directly generalisable to early-phase regional implementations. Importantly, the benefits of RAS are not uniform across all procedures or patient populations. Learning curves, procedural complexity, and institutional volume play a critical role in determining outcomes. In regional settings, cautious case selection and staged program expansion are essential to ensure patient safety and sustainable service delivery.

### Attracting and retaining surgical talent

The availability of RAS enhances the competitiveness of regional hospitals in attracting and retaining high quality and subspecialty-trained surgeons. A previous study has highlighted that male patients undergoing prostatectomy were attracted to centres offering RARP and would bypass centres without a robotic service. While direct evidence in regional Australian settings is limited at present, these findings suggest that availability of robotic services may influence patient referral patterns and institutional attractiveness. This echoes the Victorian experience: one of the main aims for Ballarat’s robot program is to attract skilled surgeons and trainees to the region [7]. By offering cutting-edge technology, regional centres become more appealing to both early-career trained and experienced surgeons seeking opportunities outside metropolitan settings [13].

This strategic effect is crucial given Australia’s rural workforce shortages. The Rural Health Equity report by RACS highlights that fewer than 5% of many specialist surgeons work outside cities [2]. If remote hospitals cannot offer modern tools, they risk falling behind in attracting talented surgeons. In fact, the recent RACS working party on robotics explicitly warned that without equitable distribution of RAS systems, rural centres will lose access to “a generation of skilled specialists who may refuse rural postings” [14]. Furthermore, literature has demonstrated that there has been a recent shift to RAS-competent surgeons, with one cohort study demonstrating 73.5% of early-career surgeons completing a robotic surgical fellowship compared to 22.2% of those with greater than 10 years’ experience [15]. Based on this data, it shows that more surgeons are becoming trained and competent in RAS, therefore rural and regional areas need to invest to recruit and retain. Installing a robotic platform regionally signals institutional commitment to cutting-edge care and surgeon development. It provides local surgical teams with professional growth, from learning the latest techniques to providing the ability to expand research capacity. Alternatively, without robotic capability, regional centres may increasingly struggle to recruit early-career surgeons, highlighting the strategic importance of investing in such technology for future service viability.

## The challenges ahead

### Cost and sustainability

Robotic surgery platforms are expensive to acquire and maintain. A typical Da Vinci ^®^ system, for example, is valued at nearly $4 million dollars, with per case consumables of roughly $1800 and annual service contracts exceeding $600,000 [13]. For rural public hospitals operating within the constraints of the federal budget, financial sustainability will remain a significant challenge. While government funding has facilitated the initial implementation at Ballarat, other rural hospitals may struggle to replicate this model without similar financial support.

Beyond the upfront costs, there are ongoing expenses associated with robotic platforms—including maintenance, instrument replacement, and system upgrades, which pose a continuous financial burden [16]. Public health policies should consider sustainable funding models to ensure that RAS remains a viable option in rural and regional Australia. Collaborations between state governments, healthcare networks, and private industry could provide potential pathways for shared-cost models that enhance accessibility [7]. Currently, Australia’s national healthcare scheme, Medicare, does not recognise the difference in surgical approach; that is, the distinctions between open, laparoscopic or robotic surgery. Funding policy and strategies should be lobbied to incorporate appropriate remuneration for the procedure type thus supporting the ongoing financial sustainability of robotics.

Opportunity cost is another significant consideration for implementation of a RAS system. In regional settings, capital directed toward robotic platforms may reduce the ability to invest in other institutional priorities, such as additional specialist staffing, critical care capacity or imaging services. Consequently, decisions regarding RAS adoption should be framed as questions of health system resource allocation rather than technology acquisition alone.

In theory, improvements in clinical outcomes may translate into downstream economic benefits over time, particularly through reduced length of stay and complication-related costs. For example, a simple bed cost analysis, based on Independent Health and Aged Care Pricing Authority data, denotes an average day bed cost as $2495.6 as per July 2023 [17], having the potential to save thousands of dollars per patient on average. A recent economic modelling study noted that even under public reimbursement, the per-patient cost of RAS is largely driven by fixed capital and service fees [18], therefore, any reduction in length of stay or complications meaningfully improves value of the robotic platform as case numbers increase. However, long term economic analyses should be performed to review the case numbers required to offset the costs of RAS system implementation. In a regional setting this means that the case volume needs to stay high enough to amortise the significant fixed capital and maintenance cost.

Further, case-mix suitability is critical to maximise the value of RAS programs in regional and rural areas. Rather than deploying robotics indiscriminately across all procedures, regional services should prioritise cases where technology offers clear technical or clinical advantages. Conversely, routine or low-complexity cases that can be performed safely and efficiently with conventional laparoscopy may not justify the additional robotic cost or resource utilisation.

In summary, extending RAS to rural hospitals offers potential clinical and system benefits, but its economic value is dependent on appropriate case selection and sufficient procedural volume.

### Education and training

Introducing robotic surgery also requires a significant investment in training [19]. Currently, Australia lacks a standardised training curriculum for RAS. The initial introduction and familiarisation of robotic platforms are largely led by manufacturers and institutions. While various training models have been proposed, none have been formally adopted into surgical training programs. A proactive, structured training framework, equally accessible to both metropolitan and regional centres, is essential to support equitable adoption of this emerging technology.

Drawing from the history of laparoscopic surgery adoption, initial efforts have focused on upskilling consultants, with the expectation that increased technology availability will lead to RAS becoming standard practice and integrating into surgical training. Lessons from the initial hesitancy surrounding laparoscopic surgery highlight the importance of early adoption of robotic technology to enable rapid upskilling and professional growth. While the clinical benefits are significant, the “learning curve” remains a substantial hurdle. Transitioning from laparoscopic to robotic platforms requires an initial period of increased operative time and potential efficiency loss [21]. For regional centres with lower case volumes, achieving the plateau of proficiency may take longer than in high-volume metropolitan hubs, requiring institutional patience and protected training time [22]. There is also a risk that early robotic adoption may concentrate operative exposure among credentialed surgeons, potentially limiting case access for trainees not embedded within robotic programs unless training pathways are deliberately integrated.

Attaining the first robot in a regional public hospital has allowed both regional surgeons and surgical trainees to gain exposure to RAS and develop necessary skills. The current training model adopted by Grampians Health requires surgeons to complete up to 20 h of simulator training, followed by proctored cases under an experienced mentor, to become certified in robotics [23]. This model combines simulation, hands-on practice and supervised live case – which reflects international best practice for surgical education techniques [24]. Grampians Health currently has ten fully trained surgeons performing robotic-assisted surgeries across multiple specialties, including colorectal, upper gastrointestinal, thoracic, urology, hernia and gynaecological surgery [6]. The institution aims to expand this number in the future in a sustainable plan.

To maintain surgical proficiency and improved patient outcomes, high patient volume and console time is crucial. It is hoped that once there is an established robotics program at a hospital, surgical trainees will be able to receive adequate hands on exposure to robotic surgery during their training [19, 25]. With 24 out of 27 public Da Vinci^®^ Platforms available in Australia being dual console systems, Australia is well placed to commence and form established robotic surgical training [4]. However, limited access to the technology in rural and regional areas remains a significant barrier to consistent RAS training across all surgical trainees [25]. Currently, most robotic cases are performed in an elective setting. Branching from elective to emergency surgery may provide more volume and opportunities for the trainees to undertake training - a lesson learnt from adopting laparoscopic surgery.

Collaboration with metropolitan centres for structured RAS fellowships and regional training hubs could help bridge the current training gap. Large tertiary centres with established RAS programs can partner with regional hospitals through “hub-and-spoke” models. Visiting surgeon programs, where city-based robotics teams travel periodically to rural centres for joint lists, can train local staff while boosting case numbers for both. Over time, as trainees in regional and rural settings become proficient, they too can act as local mentors. Such partnerships, possibly facilitated by state health departments and the Royal Australasian College of Surgeons, would help build a distributed training pipeline. Additionally, advocating for formal inclusion of RAS training in surgical curricula through professional colleges will be critical in ensuring equitable education pathways for all trainees, regardless of geographic location [5].

On a final note, minimally invasive surgery remains fundamental to upholding the high surgical standards for which Australia is recognised. As such, those shaping national healthcare policy must recognise that quality is as important as quantity. Currently Initiative’s Da Vinci^®^ robotic systems dominates the Australian robotic surgery market. However, as new entrants gain traction, such as systems from Medtronic, CMR Surgical, and others, it is anticipated that competition will drive down both consumable costs and capital investment requirements. However, there should be an onus on industry stakeholders provide not only technological advancement, but also sustainable pricing and support models tailored to the needs of Australia’s publicly funded and geographically diverse health system. Ensuring that RAS does not exacerbate healthcare inequity will require a shared commitment to affordability, access, and long-term value across all stakeholders.

### Ensuring equity in technological access

Bringing robotic surgery to rural Australia must itself be done equitably. To date, RAS availability has clustered in well-resourced areas and, without deliberate planning, underserved communities’ risk being left further behind. Policymakers should therefore articulate a national strategy for equitable RAS access. This could involve setting targets or incentives for rural hospital adoption, aligned with existing rural health commitments. For example, just as telehealth Medicare items were specifically expanded for rural patients during COVID-19 [26], similar approaches might earmark funding for rural robotics installations.

Infrastructure investment is crucial; many remote hospitals lack not only high-speed broadband, but even consistent operating theatre time blocks. Government and health services must ensure these foundational needs are met. Grants or subsidies could support the initial capital outlay for hospitals in higher remoteness categories. The Commonwealth’s “Rural Health Infrastructure Fund” or rural innovation grants could be avenues. Furthermore, establishing regional training and maintenance centres, potentially run cooperatively by a network of rural hospitals, would reduce technical barriers.

National-level coordination is also needed. A national RAS plan could map existing and planned installations, to identify gaps and avoid duplication. It should incorporate input from rural health stakeholders, ensuring Aboriginal and Torres Strait Islander communities and other marginalised groups benefit. As one expert commentary noted, addressing “urban paternalism” in health requires that advanced services be genuinely accessible to rural patients [27].

Finally, we must recognise that technology equity is part of a broader social justice effort. Enabling a regional patient to receive robotic care locally is more than a technical upgrade – it is a step towards parity. Achieving this vision will require cross-sector collaboration, stable funding commitments, and innovative models of surgical services delivery.

## Limitations

This perspective draws on the experience of a single-centre regional hospital; which may limit generalisability to other rural or regional centres with different patient populations, workforce capacity, infrastructure and fundings models. Broader applicability will require validation across multiple regional centres with differing service profiles and resource constraints. The absence of prospective design and contemporaneous open or laparoscopic comparators precludes causal attribution of observed activity to robotic-assisted surgery itself in reference to clinical outcomes, cost-effectiveness, or system impact.

Current observations reflect an early implementation phase, and longitudinal assessment is needed to characterise learning-curve effects and steady-state performance. Given the lower procedural volumes typical of regional settings, future research should explicitly examine scalability, workforce sustainability, and training models. Importantly, dedicated evaluation of patient-reported outcomes, cultural safety, and equity impacts—particularly for rural and Aboriginal and Torres Strait Islander populations—is essential to ensure regional robotic surgery advances, rather than inadvertently extending the healthcare equity gap.

## Conclusion

The introduction of RAS in rural and regional Australia represents a meaningful evolution in surgical service delivery. By expanding access to advanced surgical care, improving patient outcomes, and strengthening the rural surgical workforce, robotic surgery has the potential to meaningfully enhance healthcare delivery in these communities. Moving forward, realising the potential benefits of RAS in wider rural and regional Australia will require realistic economic appraisal, coordinate training frameworks, and equitable policy support.

## Data Availability

No datasets were generated or analysed during the current study.
